# The Role of Low-Spatial Frequency Components in the Processing of Deceptive Faces: A Study Using Artificial Face Models

**DOI:** 10.3389/fpsyg.2019.01468

**Published:** 2019-06-26

**Authors:** Ken Kihara, Yuji Takeda

**Affiliations:** Automotive Human Factors Research Center, National Institute of Advanced Industrial, Science and Technology (AIST), Tsukuba, Japan

**Keywords:** facial expression, deceptive face, spatial frequency, face-generating task, face-classification task

## Abstract

Interpreting another’s true emotion is important for social communication, even in the face of deceptive facial cues. Because spatial frequency components provide important clues for recognizing facial expressions, we investigated how we use spatial frequency information from deceptive faces to interpret true emotion. We conducted two different tasks: a face-generating experiment in which participants were asked to generate deceptive and genuine faces by tuning the intensity of happy and angry expressions (Experiment 1) and a face-classification task in which participants had to classify presented faces as either deceptive or genuine (Experiment 2). Low- and high-spatial frequency (LSF and HSF) components were varied independently. The results showed that deceptive happiness (i.e., anger is the hidden expression) involved different intensities for LSF and HSF. These results suggest that we can identify hidden anger by perceiving unbalanced intensities of emotional expression between LSF and HSF information contained in deceptive faces.

## Introduction

In our daily communication, facial expressions are one of the main cues used to understand other people’s emotions or internal states. People often try to conceal their emotions (i.e., what they are truly feeling), instead presenting an opposing or different expression (Porter et al., 2011a). Nevertheless, we do depend on understanding true emotions in order to establish good personal relationships. Thus, interpreting true emotion is important for favorable communication (King, 1998; Butler and Gross, 2004). Generally speaking, it is difficult to generate expressions that appear the same as spontaneous ones. For example, deceptive happiness expressions are distinguishable from genuine happiness expressions by observing the movements of the zygomatic major and orbicularis oculi muscles (Ekman and Friesen, 1982). In fact, observers can discriminate between genuine and deceptive facial expressions rather rapidly (Porter and Ten Brinke, 2008). The interpretation of facial expressions depends on the observer. This is because one observer might judge a face as showing genuine anger, whereas another observer might judge the same face as showing deceptive anger. However, the type of facial information that is used for interpreting another person’s hidden emotions or recognizing deceptive faces is unclear.

In this study, we focused on the spatial frequency components of faces, which are important for interpreting facial expressions (Ruiz-Soler and Beltran, 2006). Low-spatial frequencies (LSFs) carry information about the configural properties of facial parts, such as the eyes, the nose, and the mouth, whereas high-spatial frequencies (HSFs) contain finer, edge-based information supporting the processing of these features, resulting in detailed image representations and object boundaries (Goffaux et al., 2005). In studies examining the perception of static natural scenes, the parallel processing of LSF and HSF information extracted and integrated from scene images has led to the rapid interpretation of scenes presented for a short duration (i.e., 100 ms) and perceived ambiguously due to the short presentation (e.g., Kihara and Takeda, 2010, 2012). Consequently, we expected that combining different information provided by LSF and HSF would contribute to interpreting not only natural scenes presented for a short duration but also ambiguous facial expressions. Related to the above, it has been suggested that LSF and HSF components play different roles in the perception of facial expressions. Schyns and Oliva (1997, 1999) found that if face stimuli are hybrid images composed of one expression in LSF and another expression in HSF, categorizing facial expression (e.g., happiness versus anger) is dependent mainly on LSF, whereas identifying the presence of emotional expression (e.g., emotional versus neutral) is based on HSF information. Although previous findings are based on genuine-face stimuli, LSF and HSF may also contain different types of emotional cues that are used to interpret deceptive facial expressions. Indeed, characteristics of deceptive facial expressions are shown in both upper and lower face (Porter et al., 2011b), i.e., deceptive facial expression does not depend on characteristics of specific facial parts. This implies that LSF information carrying the global shape and structure of a face may play an important role in identifying deceptive facial expressions.

Several studies have identified the differential contributions of LSF and HSF to the interpretation of facial expressions. For example, Laeng et al. (2010) used hybrid images composed of emotional expressions in LSF and neutral expression in HSF and demonstrated that LSF plays an essential role in the implicit detection of emotional expressions. The participants in their study rated the images as friendly or unfriendly based on the LSF component, whereas they explicitly judged the images as neutral regardless of the LSF component. Importantly, Prete et al. (2014, 2015c) reported hemispheric asymmetry in neural processing for implicit detection of emotional expressions because hybrid images tend to be rated as less friendly when they are presented in the left visual field than in the center or the right visual fields (see also Prete et al., 2018b, for a transcranial stimulation study). This tendency is also shown when unfiltered, intact images are used as the to-be-rated expressions (Prete et al., 2015b). Also, both hybrid and intact images cause emotional aftereffects in that presenting a negative expression causes the perception of subsequent neutral expressions to be judged more positively and vice versa (Prete et al., 2018a). Furthermore, an event-related potentials study has indicated that facial and emotional processing-related P1, N170, and P2 components are evoked by hybrid, as well as intact images (Prete et al., 2015a). Such evidence suggests that hemispheric asymmetry for implicit emotional processing is a robust phenomenon that is not limited to the specific use of hybrid images. Interestingly, sensitivity for the implicit detection of emotional expressions is enhanced after oxytocin treatment as reflected by pupilar dilation because of the allocation of attention to socially relevant information (Leknes et al., 2013). These findings clearly suggest that LSF but not HSF component contributes to the implicit perception of emotional faces, implying that the perception of deceptive facial expressions, which might be processed intuitively and implicitly, is affected by LSF rather than HSF component.

It has also been demonstrated that LSF and HSF do not equally contribute to identifying negative expressions (Vuilleumier, 2005). Understanding negative emotion from facial expressions is potentially important for survival (relative to positive emotion), and this may be why the visual system is biased toward the processing of negative expressions (Taylor, 1991). In fact, negative expressions attract and hold attention more frequently and for longer than positive expressions (Mathews et al., 1997). Importantly, the prioritized processing of negative expressions is specifically attributed to the neural pathway tuned to LSF components (Vuilleumier, 2005). Low-spatial frequency preserves coarse information associated with an object’s shape and layout, which is transmitted to the cortex and subcortical structures through the rapid magnocellular pathways (Bar, 2004). A functional magnetic resonance imaging (fMRI) study showed that the human amygdala, which allocates attention to negative stimuli (LeDoux, 1995), selectively responds to the LSF, but not the HSF, component of fearful expressions (Vuilleumier et al., 2003). This result suggests that LSF information projected to amygdala *via* the magnocellular pathways plays an important role in processing negative expressions (Winston et al., 2003; Pourtois et al., 2005; but see also Holmes et al., 2005; Morawetz et al., 2011, for different results). It is therefore possible that the involvement of LSF components differs when viewing deceptive faces hiding negative versus positive emotional states.

The current study investigated whether LSF or HSF components are more important when interpreting deceptive faces. To address this issue, we examined the intensity of the emotional expressions contained in LSF and HSF components of deceptive faces using two different tasks. It is known that dynamic elements of faces are critical for interpreting facial expressions (Krumhuber et al., 2016) because perception of dynamic elements is asymmetrically processed in LSF and HSF (Kauffmann et al., 2014). However, in this study, we focused on static situations for investigating the basic role of spatial frequency information on interpreting deceptive expressions. In Experiment 1, we used a face-generating task where participants were asked to generate genuine and deceptive faces by tuning the intensity of specific expressions in both LSF and HSF. There were two genuine faces: genuine happiness (positive) and genuine anger (negative). There were also two deceptive faces: deceptive happiness (concealing genuine anger) and deceptive anger (concealing genuine happiness). In Experiment 2, we used a face-classification task where participants were asked to classify presented faces composed of LSF and HSF expressions as either genuine happiness, genuine anger, deceptive happiness, or deceptive anger. Because the LSF component is important for discriminating positive versus negative facial expressions (Schyns and Oliva, 1997, 1999), we assumed that identifying the hidden expression would depend on the intensity of the expression represented in LSF. We predicted that interpreting hidden negative emotion would depend heavily on the LSF component of the deceptive positive faces, because LSF information plays a critical role in the preferential processing of negative expressions (Vuilleumier, 2005). It is important to note that we used artificial facial models because we had to control the intensity of facial expressions step by step. In addition, the artificial facial models allow us to make different facial expressions with minimal changes of each individual faces, which should be critical to superimposing two images consisting of different spatial frequencies as an integrated one.

## Experiment 1

### Materials and Methods

#### Participants

Twenty-seven adult males (mean age 23, range 19–31) from the subject pool at the National Institute of Advanced Industrial Science and Technology participated in this experiment. All participants received payment for their participation. All had normal or corrected-to-normal vision and were right-handed. This study was carried out in accordance with the recommendations of Guidelines for handling ergonomic experiments, Committee on Ergonomic Experiments, Bioethics and Biosafety Management Office, Safety Management Division, National Institute of Advanced Industrial Science and Technology and approved by the Committee on Ergonomic Experiments, Bioethics and Biosafety Management Office, Safety Management Division, National Institute of Advanced Industrial Science and Technology. All participants gave written informed consent in accordance with the Declaration of Helsinki.

#### Stimuli and Apparatus

**Figure 1:**
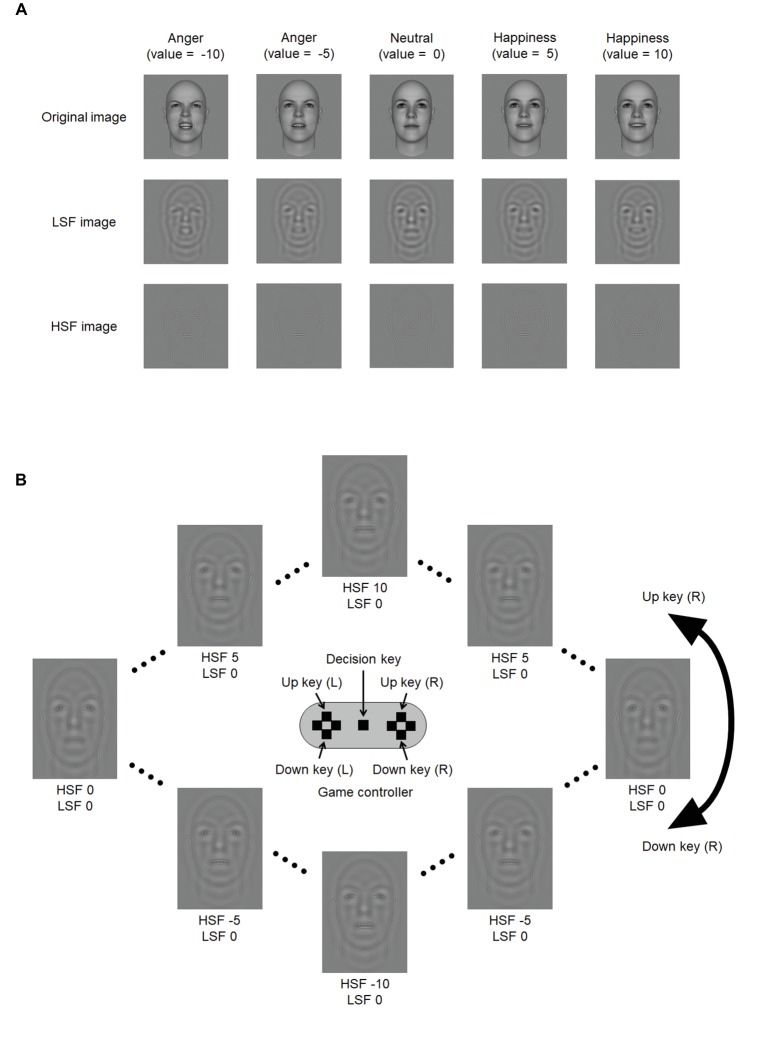
Example of a randomly generated face image by FaceGen Modeller 3.5 software and schematic illustrations of the procedure. **(A)** Example images in the two frequency conditions and the original image. In the experiment, 21 variations in expression from the angriest (expression value of −10) to the most happiness (expression value of 10) were used. LSF images were filtered with low band-pass frequencies (4–8 c/f). HSF images were filtered with a high band-pass filter (16–32 c/f). **(B)** Schematic illustration of the game controller and the relationship between the up and down keys used to change the expression value of the image. In this schematic, the up key on the right side of the controller changes the HSF component of the image, ranging between −10 and 10 (in single steps) in a counterclockwise direction. The down key changes the HSF component of the image in the opposite direction. The up and down keys on the left side of the controller change the LSF component of the image.

Examples of facial images are given in Figure 1A. Eighty individual faces in frontal view were randomly generated using FaceGen Modeller 3.5 (Singular Inversions Inc.). FaceGen Modeller software allows us to manipulate realistic facial expressions, which are available for a wide range of facial expression studies (e.g., Corneille et al., 2007; Schulte-Rüther et al., 2007; Oosterhof and Todorov, 2008; Xiao et al., 2015; Hass et al., 2016). Faces were randomized for gender, age, race, and features (brow ridge, cheekbones, etc.). Each face was morphed from neutral to happy (positive) and angry (negative) in 10 steps of increasing intensity. Thus, there were 21 variations in expression, including the neutral expression, for each individual face. These expressions were given values of from −10 (the most angry) to 10 (the most happy). The neutral face was given a value of zero. The resolution of the images was 400 × 400 pixels, which subtended 6° of visual angle at a viewing distance of about 57 cm. The width of each face was about half the size of the image width. All images were converted into grayscale LSF and HSF images. The ranges of band-pass frequencies for LSF and HSF were selected based on previous studies (Schyns and Oliva, 1999; Vuilleumier et al., 2003). The LSF images were filtered in Fourier space, using a fourth-order Butterworth filter, set to filter low band-pass frequencies (1.33–2.67 cycle/degree). The HSF images were filtered with a fourth-order Butterworth high band-pass filter (5.33–10.67 cycle/degree).

#### Procedure

There were four conditions, such as genuine happiness, genuine anger, deceptive happiness, and deceptive anger, presented in separate blocks of trials. The 80 individual faces were randomly assigned to each block, 20 faces in each. Block order was randomized. Each participant completed a total of 80 trials (4 conditions in separate blocks × 20 individual faces). Before the experiment began, participants completed eight practice trials, using different faces that were not part of the experimental trials. The experiment was conducted in a darkened room and took about 30 min to complete.

Each block began with instructions as to which face type was to be generated: “Please generate the following expression” and then “Genuine happiness,” “Genuine anger,” “Happiness but actually anger,” or “Anger but actually happiness” in Japanese. The instruction remained displayed until the central key on the game controller (designated as the decision key) was pressed. Subsequently, a randomly selected face was presented at the center of the display with the face type to-be-generated displayed below. Each face was created by averaging LSF and HSF images, both of which were randomly selected from the 21 variations of each individual face. Participants were asked to generate the instructed expression by pressing the designated keys on the game controller. For example, the up and down keys on the left side were designated to change the LSF image, and the keys on the right side changed the HSF image. The up and down keys changed the expression value of the image in opposite directions by one step. The changes were continuous; when a maximum value was reached and the same key was pressed, the value of the expression began to decrease. Similarly, when a minimum value was reached, the value of the expression began to increase. See Figure 1B for a schematic illustration. The assignment of the keys (i.e., left- and right-hand side of the game controller) was counterbalanced across participants. Participants were allowed to generate each face at their own pace. They pressed the decision key when they were finished, after which the next face appeared.

### Results and Discussion

**Figure 2:**
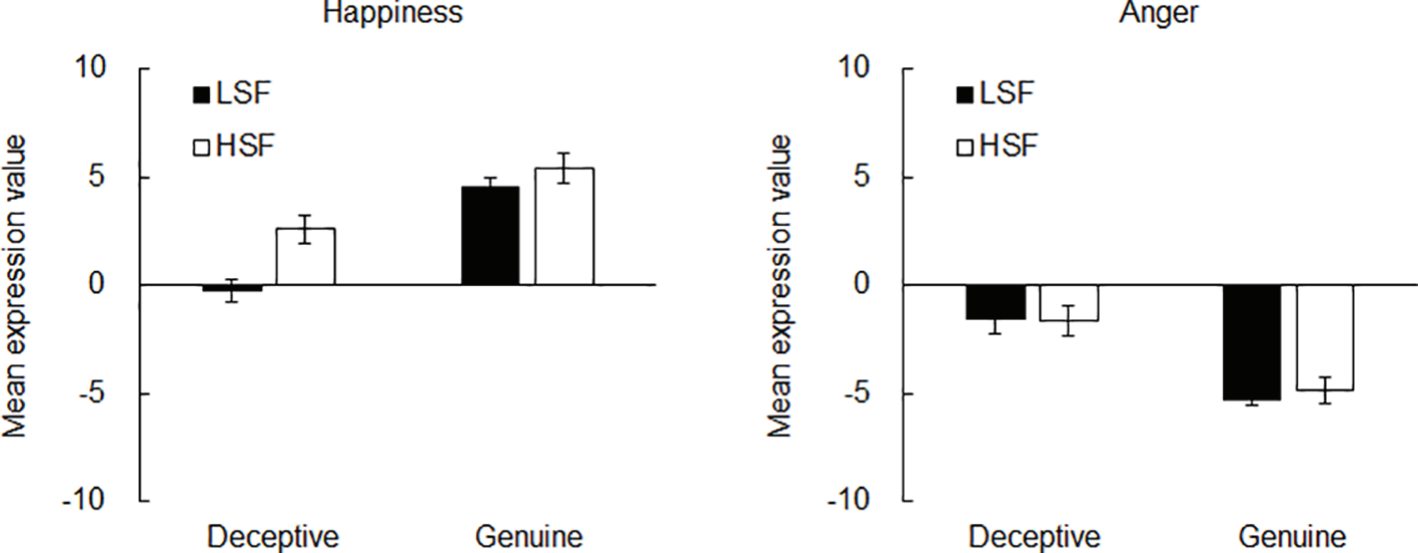
Results of Experiment 1. Mean expression value of the deceptive and genuine images created for each frequency in the happiness (left panel) and anger (right panel) conditions. Error bars indicate standard errors of the mean.

Figure 2 shows the mean expression value of the images developed for each spatial frequency in the happiness and anger blocks. In this study, although a three-way analysis of variance (ANOVA) with emotion (happiness or anger), face to-be-generated (deceptive or genuine), and spatial frequency as independent variables could be preferred to prevent a possible loss of effects, we conducted two-way ANOVAs separately for the happiness and anger blocks because the meaning of values in the happiness and anger blocks could be in opposite direction. That is, a lower value in the happiness block indicates the facial expressions close to neutral, whereas a lower value in the anger block indicates more negative facial expressions. On the other hand, the biases toward positive (negative) facial expressions can result in higher (lower) values both in the happiness and anger blocks. In this case, the interpretation of the three-way ANOVA can be very complex. Therefore, we decided to use two-way ANOVAs separately for the happiness and anger blocks. The independent variables (within-subject factors) were face to-be-generated (deceptive or genuine) and spatial frequency. The dependent variable was the mean expression value. The ANOVA revealed that there was a significant main effect of the face to-be-generated when the expression was happiness, *F*(1, 26) = 74.77, *p* < 0.001, $\eta_{p}^{2}=0.74$. The power of the *post hoc* analysis calculated by G-power 3.1.9 (Faul et al., 2007, 2009) = 1.00. The mean values (±SD) of the deceptive and genuine conditions were 1.16 (±3.29) and 4.97 (±3.04). There was also a significant main effect of the spatial frequency, *F*(1, 26) = 7.77, *p* < 0.01, $\eta_{p}^{2}=0.23$, power = 1.00. The mean values (±SD) were 2.14 (±3.43) for LSF and 3.98 (±3.73) for HSF. Importantly, there was a significant interaction between the face to-be-generated and the spatial frequency, *F*(1, 26) = 5.74, *p* < 0.03, $\eta_{p}^{2}=0.18$, power = 1.00. *Post hoc* analysis using the Duncan test (*p* < 0.05) revealed that there were significant differences between all the conditions except between the LSF and HSF conditions in the genuine face condition. The mean values (±SD) were −0.27 (±2.61) for LSF-deceptive, 2.59 (±3.31) for HSF-deceptive, 4.55 (±2.27) for LSF-genuine, and 5.38 (±3.65) for HSF-genuine. These results suggest that genuine happiness contains equally high intensity LSF and HSF components (i.e., approximately five points of mean expression values each), whereas deceptive happiness consists of differential intensities of expression in terms of LSF (i.e., approximately zero points) and HSF (i.e., approximately three points). Conversely, the ANOVA for the angry faces revealed a significant main effect of the face to-be-generated, *F*(1, 26) = 61.90, *p* < 0.001, $\eta_{p}^{2}=0.70$, power = 1.00 (deceptive: −1.62 ± 3.69; genuine: −5.08 ± 2.43). However, there was no significant main effect of the spatial frequency, *F*(1, 26) = 0.04, *p* > 0.84, $\eta_{p}^{2}=0.01$, power = 0.07. The mean values (± SD) were −3.42 (±3.40) for LSF and −3.27 (±3.74) for HSF. Also, there was no significant interaction, *F*(1, 26) = 0.15, *p* > 0.69, $\eta_{p}^{2}=0.01$, power = 0.17. The mean values (± SD) were −1.58 (± 3.72) for LSF-deceptive, −1.66 (± 3.72) for HSF-deceptive, −5.27 (± 1.63) for LSF-genuine, and −4.89 (± 3.05) for HSF-genuine. These results suggest that deceptive anger is different from genuine anger only in terms of the intensity of anger expressed, regardless of spatial frequency.

## Experiment 2

The results of Experiment 1 demonstrated that only deceptive happiness consisted of differential expression intensities for LSF and HSF. These findings were provided by a face-generation task in which participants generated the instructed facial expressions. To validate these results independently of task demands, we next examined whether the findings from the face-generation task could be replicated using another task. In Experiment 2, we used a face-classification task in which participants were asked to classify presented faces that depicted certain LSF and HSF expression values as either genuine happiness, genuine anger, deceptive happiness, or deceptive anger. We predicted that Experiment 2 would produce a similar pattern of results to Experiment 1, if indeed differential intensities of expression between LSF and HSF are an important cue for interpreting deceptive happiness. That is, the faces showing lower LSF expression as compared to HSF would tend to be classified as deceptive happiness.

### Materials and Methods

#### Participants

Thirty-three adult males (mean age 22.4, range 18–34) from the subject pool at National Institute of Advanced Industrial Science and Technology participated in this experiment. All participants received payment for their participation. All had normal or corrected-to-normal vision and two were left-handed. This study was carried out in accordance with the recommendations of Guidelines for handling ergonomic experiments, Committee on Ergonomic Experiments, Bioethics and Biosafety Management Office, Safety Management Division, National Institute of Advanced Industrial Science and Technology and approved by the Committee on Ergonomic Experiments, Bioethics and Biosafety Management Office, Safety Management Division, National Institute of Advanced Industrial Science and Technology. All participants gave written informed consent in accordance with the Declaration of Helsinki.

#### Stimuli, Apparatus, and Procedures

Stimuli, apparatus, and procedures were the same as those used in Experiment 1, except for the changes described here. Twenty individual faces were randomly chosen from the pool of 80 individual faces used in Experiment 1. There were five variations of expression value for both LSF and HSF images for each individual face (i.e., expression values are −10, −5, 0, 5, and 10). To-be-classified faces were created by averaging LSF and HSF images, both of which were selected from the five variations of each individual face. All possible combinations of LSF and HSF images were used. Thus, 500 faces (20 individuals × 5 values in LSF × 5 values in HSF) were used for the classification task.

At the start of the experiment, a randomly selected face was presented at the center of the display. After 2,000 ms, participants were asked to classify the presented face as “Genuine happiness,” “Genuine anger,” “Happiness but actually anger,” or “Anger but actually happiness” by pressing the designated keys on the game controller, without time pressure. After pressing the key, the next face appeared. Face order was randomized. Each participant completed a total of 500 trials. Before the experiment began, participants completed eight practice trials, using different faces that were not used during the experimental trials. The experiment was conducted in a darkened room and took about 40 min to complete.

### Results and Discussion

**Figure 3:**
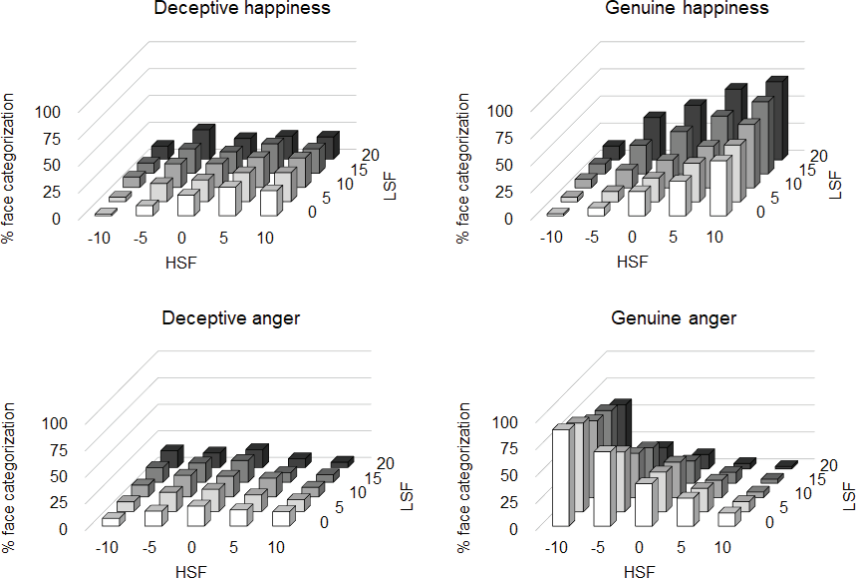
Results of Experiment 2. Mean percentage of classification as the deceptive happiness (top-left panel), the genuine happiness (top-right panel), deceptive anger (bottom-left panel), and genuine anger (bottom-right panel).

Figure 3 shows the mean classification percentages for the four types of face across participants. Obviously, participants tended to classify the faces comprising higher expression values for both LSF and HSF as genuine happiness. Conversely, they classified the faces with lower expression values for both LSF and HSF as genuine anger. On the contrary, zero or near zero values for both LSF and HSF seem to be preferred as the deceptive faces.

**Figure 4:**
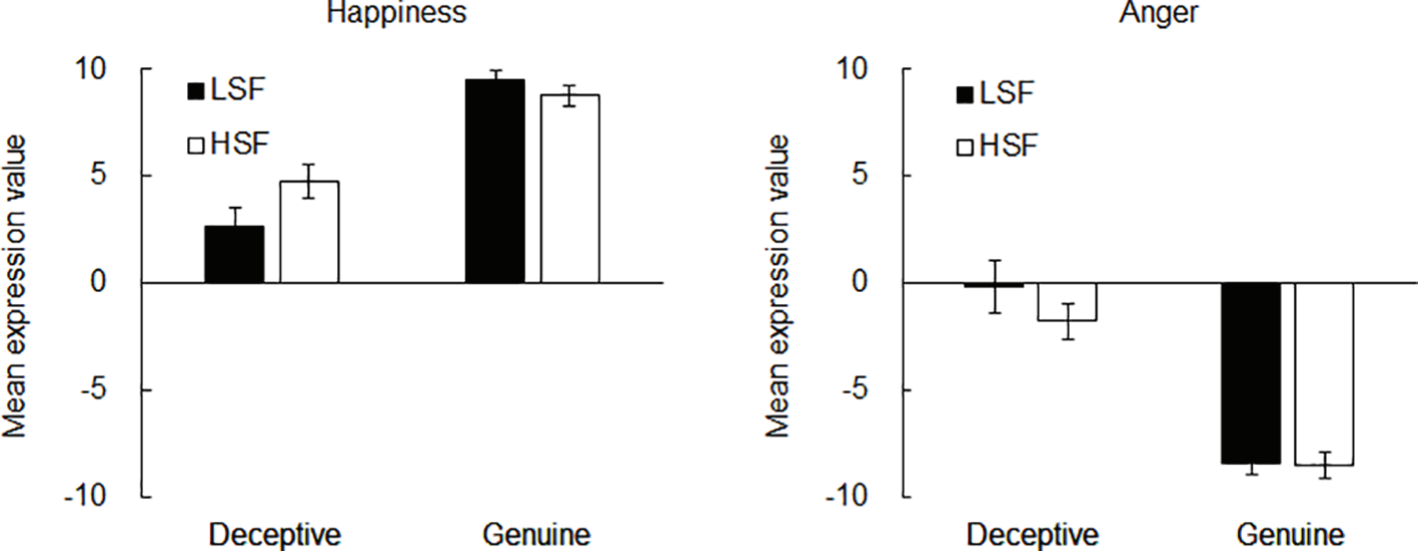
Results of Experiment 2. Mean expression values of the mode for the deceptive and genuine images created by each frequency in the happiness (left panel) and anger (right panel) expressions. Error bars indicate standard errors of the mean.

We estimated the mode of the data of each participant to clarify the combination of LSF and HSF expression values that were subject to be classified as each face type. If the mode was more than one (i.e., there were two or more peaks in the frequency histogram), they were averaged. We decided to use the mode rather than the mean of the classification proportion because the mean would not reflect the typical values in each category. For example, typical values of genuine anger expression should be near −10 for LSF and HSF. However, the mean values of the classification proportion increase close to zero because of the edge effect (i.e., stimuli more negative than −10 cannot be made). Therefore, the mode was considered appropriate to estimate the typical values in each category. Figure 4 shows the mean expression value of the mode for each spatial frequency for happiness and anger expressions across the participants. A two-way ANOVA with the mean expression value of the mode as the dependent variable indicated that there was a significant main effect of the face for the expression of happiness, *F*(1, 32) = 52.95, *p* < 0.001, $\eta_{p}^{2}=0.62$, power = 1.00. The mean values (±SD) were 3.72 (± 4.96) for deceptive and 9.17 (± 2.53) for genuine. However, there was no significant main effect of the spatial frequency, *F*(1, 32) = 2.38, *p* > 0.13, $\eta_{p}^{2}=0.07$, power = 0.87. The mean values (±SD) were 6.11 (±5.22) for LSF and 6.78 (±4.31) for HSF. Importantly, there was a significant interaction between the face and the spatial frequency, *F*(1, 32) = 5.42, *p* < 0.03, $\eta_{p}^{2}=0.14$, power = 1.00. *Post hoc* analysis using the Duncan test (*p* < 0.05) revealed that there were significant differences between all the conditions except between LSF and HSF in the genuine face condition. The mean values (± SD) were 2.68 (± 5.11) for LSF-deceptive, 4.77 (± 4.65) for HSF-deceptive, 9.55 (± 2.21) for LSF-genuine, and 8.79 (± 2.80) for HSF-genuine. The ANOVA for the angry faces revealed a significant main effect of the face, *F*(1, 32) = 55.58, *p* < 0.001, $\eta_{p}^{2}=0.63$, power = 1.00. The mean values (±SD) were −0.95 (±6.01) for deceptive and −8.45 (±3.30) for genuine. However, there was no significant main effect of the spatial frequency, *F*(1, 32) = 1.04, *p* > 0.31, $\eta_{p}^{2}=0.03$, power = 0.54. The mean values (±SD) were −4.28 (±6.78) for LSF and −5.11 (±5.41) for HSF. Also, there was no significant interaction, *F*(1, 32) = 0.96, *p* > 0.33, $\eta_{p}^{2}=0.03$, power = 0.64. The mean values (±SD) were −0.15 (±7.01) for LSF-deceptive, −1.74 (±4.78) for HSF-deceptive, −8.41 (±2.99) for LSF-genuine, and −8.48 (±3.64) for HSF-genuine. These results are consistent with those of Experiment 1. The finding suggests that deceptive happiness consisted of differential expression intensities for LSF and HSF does not depend on the task demands.

## Discussion

The spatial frequency components of faces provide critical clues for recognizing facial expressions (Farah et al., 1998; Ruiz-Soler and Beltran, 2006). However, it is not clear how we use such spatial frequency information in deceptive faces to interpret true emotion, and whether the contribution of LSF and HSF differs between deceptive happiness and anger facial expressions. To address these issues, we asked participants to generate deceptive and genuine faces by tuning the intensities of happiness and anger, which were contained in both LSF and HSF components (Experiment 1), and to classify presented faces composed of LSF and HSF images as either genuine happiness, genuine anger, deceptive happiness, or deceptive anger (Experiment 2). The results of the experiments show that deceptive happiness consists of differential intensities of expression between LSF and HSF, while deceptive anger consists of low LSF and HSF intensities. These results suggest that contribution of the LSF and HSF components are not equal when interpreting happy and angry deceptive faces.

The present study suggests that it is possible to discriminate deceptive happiness from a genuine one. This is because a deceptive happiness consists of unbalanced amounts of LSF and HSF expression, whereas a genuine happiness consists of approximately equal LSF and HSF intensities. In other words, detecting the unbalanced intensities of happiness expression between LSF and HSF allows us to be sensitive to hidden anger. Conversely, it must be difficult to distinguish between deceptive and genuine anger because both are represented by approximately equal LSF and HSF intensities. Although deceptive anger has lower intensities of both LSF and HSF expressions, there is no way to distinguish this from slight anger. In this case, other clues, such as facial movement, tone of voice, and/or contextual information, must be used when trying to interpret true emotion from anger facial expressions. Considering the fact that a high sensitivity for negative expressions has an adaptive function that promotes survival (Mathews et al., 1997), the visual system is likely biased toward hidden, as well as genuine, negative emotion. Based on this notion, LSF components play a key role in the sensitivity of interpreting hidden anger in deceptive happiness. Low-spatial frequency information about genuine anger facial expressions conveyed through the rapid magnocellular pathway reaches and activates the amygdala, a specific brain region for processing bias toward negative stimuli (Vuilleumier, 2005), which is essential for an adaptive function of quick risk aversion (Taylor, 1991). It is possible that the sensitivity to hidden anger in deceptive happiness found in this study is governed by the same visual pathway, although there is not yet empirical evidence for a relationship between amygdala activation and the processing of hidden anger facial expressions.

We adopted a face-generating task in Experiment 1 and a face-classification task in Experiment 2 and asked the participants to encode and decode facial expressions. Although these tasks examined different processes (i.e., encoding/decoding deceptive facial expressions), both tasks showed similar results with a trend for only deceptive happiness to show differential intensities in the expressions between LSF and HSF. These consistent results supported the notion that processing deceptive happiness depends on the balance between LSF and HSF components.

Note that we used only anger as a negative emotional expression in this study, although there are a variety of negative expressions, such as fear, disgust, and sadness. Regarding this, many studies have provided strong support for the idea that LSF components convey important information for processing of fear expressions (Vuilleumier et al., 2003; Winston et al., 2003; Pourtois et al., 2005; Vlamings et al., 2009; Bannerman et al., 2012; but see Holmes et al., 2005; Morawetz et al., 2011). It has also been suggested that LSF components of fear and disgusted expressions are related to non-conscious processing of negative expressions (Willenbockel et al., 2012). However, there are a few studies that demonstrate a relationship between HSF components and identification of grimacing (Deruelle et al., 2008) and sadness (Kumar and Srinivasan, 2011). Thus, we do not claim that the LSF component of deceptive faces is important for interpreting all hidden negative expressions. It is also possible that spatial frequency components higher than those used in this study could contain clues to identifying negative expressions. Obviously, further studies are required to investigate whether interpreting all types of hidden negative expressions is dependent on LSF components and that higher spatial frequency components contribute to discriminating between deceptive and genuine happiness.

Another limitation of the present study using artificial face models is that perceptual sensitivity to spatial frequency may differ between artificial and real faces. It has been reported that artificial facial models could give us different impression comparing with photos of real faces, although general tendencies to evaluate face images are similar (Crookes et al., 2015; Balas and Pacella, 2017; Balas et al., 2018; González-Álvarez and Cervera-Crespo, 2019). It is also unclear whether LSF and HSF components contain different facial expression when deceptive faces are made in real situations. Although our data clearly demonstrate that human observers have an ability to categorize deceptive happiness of artificial face models depending on the mismatch between LSF and HSF components, it is the first step to understand how spatial frequency information is used to identify real deceptive faces.

The results of this study are based only on male participants because of limitations in the subject pool that was available to us. Several studies have suggested that women are more sensitive to emotional faces than men (e.g., Kato and Takeda, 2017). However, many other studies have indicated that the gender of the participants does not affect the detection of emotions in hybrid facial images composed of emotional expressions in LSF and neutral expressions in HSF. For instance, Prete et al. (2014) demonstrated that female faces tend to be evaluated as more friendly than male faces regardless of LSF expression, whereas the friendliness ratings were not significantly different between male and female participants (see also Prete et al., 2015c, 2018a). Consequently, it is possible that female participants would also produce similar trends to those shown by the male participants in this study. Nevertheless, further work is needed to clarify the relationship between the gender of participants and the perception of hybrid facial expressions.

In conclusion, this study provides evidence that the LSF components of a deceptive happiness may allow us to interpret the true emotional state of anger. This finding indicates that we can understand another’s hidden anger facial expression rapidly simply by using visual information from a static face, such as the unbalanced intensities of emotional expression between LSF and HSF. On the other hand, it is difficult to distinguish between genuine and deceptive anger from faces alone, suggesting that other clues need to be used to determine the true emotion. A high sensitivity for hidden anger facial expression could contribute to an adaptive function of risk aversion.

## Ethics Statement

This study was carried out in accordance with the recommendations of “Guidelines for handling ergonomic experiments, Committee on Ergonomic Experiments, Bioethics and Biosafety Management Office, Safety Management Division, National Institute of Advanced Industrial Science and Technology”; with written informed consent from all subjects. All subjects gave written informed consent in accordance with the Declaration of Helsinki. The protocol was approved by the “Committee on Ergonomic Experiments, Bioethics and Biosafety Management Office, Safety Management Division, National Institute of Advanced Industrial Science and Technology.”

## Author Contributions

KK and YT contributed to the conception and the design of the study. YT collected the data. KK wrote the first draft of the manuscript. KK and YT read and approved the final manuscript.

### Conflict of Interest Statement

The authors declare that the research was conducted in the absence of any commercial or financial relationships that could be construed as a potential conflict of interest.
